# Adherence to the cardiac surgery checklist decreased mortality at a teaching hospital: A retrospective cohort study

**DOI:** 10.1016/j.clinsp.2022.100048

**Published:** 2022-05-17

**Authors:** Omar Asdrúbal Vilca Mejia, Frederico Carlos Cordeiro de Mendonça, Lucimar Aparecida Barrense Nogueira Sampaio, Filomena Regina Barbosa Gomes Galas, Mauricio Franklin Pontes, Luiz Fernando Caneo, Luís Roberto Palma Dallan, Luiz Augusto Ferreira Lisboa, João Fernando Monteiro Ferreira, Luís Alberto de Oliveira Dallan, Fabio Biscegli Jatene

**Affiliations:** a Quality and Safety Surgical Unit, Instituto do Coração, Hospital das Clínicas, Faculdade de Medicina, Universidade de São Paulo (HCFMUSP), São Paulo, SP, Brazil; b Department of Cardiopneumology, Instituto do Coração, Hospital das Clínicas, Faculdade de Medicina, Universidade de São Paulo (HCFMUSP), São Paulo, SP, Brazil; c Nursing Unit of the Surgical Center, Instituto do Coração, Hospital das Clínicas, Faculdade de Medicina, Universidade de São Paulo (HCFMUSP), São Paulo, SP, Brazil; d Anesthesiology Unit, Instituto do Coração, Hospital das Clínicas, Faculdade de Medicina, Universidade de São Paulo (HCFMUSP), São Paulo, SP, Brazil; e InnovaSpace UK, Space & Extreme Environment Research Center, Universidade Federal de Ciências da Saúde de Porto Alegre, RS, Brazil; f Patient Safety Subcommittee, Instituto do Coração, Hospital das Clínicas, Faculdade de Medicina, Universidade de São Paulo (HCFMUSP), São Paulo, SP, Brazil

**Keywords:** Checklists, Quality improvement, Cardiac surgery, Mortality

## Abstract

**Objective:**

To evaluate the impact of adherence to the cardiac surgical checklist on mortality at the teaching hospital.

**Methods:**

A retrospective cohort study after the implementation of the cardiac surgical safety checklist in a reference hospital in Latin America. All patients undergoing coronary artery bypass surgery and/or heart valve surgery from 2013 to 2019 were analyzed. After the implementation of the project InCor-Checklist “Five steps to safe cardiac surgery” in 2015, the correlation between adherence and completeness of this instrument with surgical mortality was assessed. The EuroSCORE II was used as a reference to assess the risk of expected mortality for patients. Cross-sectional questionnaires were during the implementation of the InCor-Checklist. To perform the correlation, Pearson's coefficient was calculated using R software.

**Results:**

Since 2013, data from 8139 patients have been analyzed. The average annual mortality was 5.98%. In 2015, the instrument was used in only 58% of patients; in contrast, it was used in 100% of patients in 2019. There was a decrease in surgical mortality from 8.22% to 3.13% for the same group of procedures. The results indicate that the greater the checklist use, the lower the surgical mortality (*r* = 88.9%). In addition, the greater the InCor-Checklist completeness, the lower the surgical mortality (*r* = 94.1%).

**Conclusion:**

In the formation of the surgical patient safety culture, the implementation and adherence to the InCor-Checklist “Five steps to safe cardiac surgery” was associated with decreased mortality after cardiac surgery.

## Highlights

• Checklists avoid human errors and are commonly used in high-reliability industries.
• The “InCor Checklist” was associated with decreased mortality over time.
• Adherence, completeness, and sustainability within public policies are necessary.

## Introduction

In the health system, adverse events cause more deaths annually than road traffic accidents, breast cancer, or acquired immunodeficiency syndrome.^1^ Global estimates suggest that every year 1 million people die after surgery. A scenario in which 50% of deaths would be preventable.^2^ Checklists began to be used in aviation in the 1930s to avoid human errors and are currently common in high-risk industries.^3^

In this way, safety checklists have been incorporated as an essential part of a safety culture.^4^ However, while surgery has continued to increase, little progress has been made in patient safety.

In 2009, World Health Organization (WHO) led the development of a checklist for surgery.^5^ The results were striking showing that the instrument was able to reduce mortality by up to 47%.^6^ The benefits seem to be directly related to an improvement in the team's communication and situation awareness, just before starting the procedure. It did not take long for the American and European Society for Cardio-Thoracic Surgery (EACTS) to consider the checklist as a class I recommendation to be applied in all cardiac surgeries.^7^^,^^8^

The morbidity and mortality after cardiac surgery decreased over time; however, avoidable errors persisted causing undesirable results. It is a complex scenario that involves sophisticated techniques and equipment, cardiopulmonary bypass, several professionals, and especially, high-risk patients. Thus, adverse events can occur due to both communication failures and lack of teamwork.^9^

Seeking to build a surgical patient safety culture, the hospital team adapted and structured its safety verification system, the InCor-Checklist “Five steps to safe cardiac surgery”. The aim of this publication is to describe the implementation and adherence of the InCor-Checklist over time, as well as its association with the mortality results after cardiac surgery.

## Methods

### Study population, inclusion, and exclusion criteria

In this analysis, 8139 patients who underwent coronary artery bypass grafting and/or heart valve surgery from 2013 to 2019 were analyzed. In total, 5 types of procedures were analyzed: coronary artery bypass graft surgery, aortic valve surgery, mitral valve surgery, aortic valve + mitral valve surgery, and coronary + heart valve surgery. Clinical Characteristics and preoperative parameters of the population analyzed are detailed in Table 1. Therefore, most surgeries were analyzed except for congenital diseases, aortic diseases, cardiac tumors, pericardial diseases, heart transplants, catheter therapies, pacemaker implantation, and patients who had salvage cardiac surgery.

**Table 1:** Characteristics of the population analyzed: clinical and pre-operative parameters.

	2013		2014		2015		2016		2017		2018		2019	
Variables	n	%	N	%	n	%	n	%	n	%	n	%	n	%
**Procedure**^a^														
CABG	584	51.1%	614	51.0%	622	52.4%	549	51.7%	523	52.2%	554	51.6%	700	47.6%
CABG + valve surgery	87	7.61%	89	7.04%	61	5.13%	40	3.77%	67	6.69%	46	4.28%	91	6.19%
Mitral valve surgery	212	18.5%	234	19.4%	255	21.5%	225	21.2%	187	18.7%	198	18.4%	374	25.5%
Aortic valve surgery	211	18.5%	215	17.9%	187	15.7%	193	18.2%	166	16.6%	211	19.6%	215	14.6%
Mitral + aortic valve surgery	49	4.29%	51	4.24%	63	5.03%	54	5.09%	58	5.79%	65	6.05%	89	6.06%
**Procedure status**														
Elective	1015	88.8%	1057	87.9%	1046	88.0%	787	74.2%	734	73.3%	778	72.4%	1125	76.6%
Urgency	128	11.2%	146	12.1%	142	11.9%	274	25.8%	267	26.7%	296	27.6%	344	23.4%
**Age, median (IQR)**^a^	63	(54‒70)	63	(54‒70)	62	(54‒69)	61	(52‒68)	62	(54‒69)	62	(53‒69)	61	(53‒68)
**Gender**														
Male	704	61.6%	762	63.3%	726	61.1%	670	63.1%	616	61.5%	648	60.3%	883	60.1%
Female	438	38.3%	441	36.7%	462	38.9%	391	36.8%	385	38.5%	426	39.6%	585	39.8%
**Systemic arterial hypertension**	389	34.0%	460	38.2%	487	41.0%	381	35.9%	377	37.7%	357	33.2%	526	35.8%
**Insulin-dependent diabetes**	26	2.27%	16	1.33%	19	1.06%	17	1.06%	19	1.09%	19	1.77%	32	2.18%
**Non-insulin dependent diabetics**	133	11.6%	116	9.64%	109	9.18%	102	9.61%	127	12.7%	106	9.87%	153	10.4%
**Acute renal failure**	11	0.96%	11	0.91%	14	1.18%	26	2.45%	25	2.05%	23	2.14%	27	1.84%
**Chronic renal failure**	24	2.1%	16	1.33%	13	1.09%	19	1.79%	14	1.04%	17	1.58%	29	1.97%
**Atrial fibrillation**	60	5.25%	61	5.07%	52	4.38%	36	3.39%	32	3.02%	32	2.98%	53	3.61%
**Obesity (IMC >40)**	29	2.54%	28	2.33%	26	2.19%	20	1.89%	20	1.99%	24	2.23%	34	2.31%
**LVEF, median (IQR)**^a^	60	(50‒64)	61	(55‒64)	61	(55‒64)	61	(55‒64)	61	(55‒64)	61	(55‒65)	60	(55‒64)
**EuroSCORE, mean, ± standard deviation**	1.98	±1.13	1.51	±0.53	2.53	±2.92	2.72	±4.58	2.52	±4.22	2.51	±4.23	2.48	±4.31
**New York Heart Association NYHA**														
I	8	0.69%	7	0.58%	9	0.76%	6	0.57%	6	0.06%	6	0.56%	8	0.54%
II	1126	98.5%	1189	98.8%	1169	98.4%	1033	97.4%	972	97.1%	1054	98.1%	1436	97.7%
III	6	0.52%	7	0.58%	8	0.67%	19	1.79%	20	2.00%	12	1.12%	19	1.29%
IV	3	0.26%	0	0%	2	0.17%	3	0.28%	3	0.03%	2	0.19%	6	0.41%
**ASA score**^b^														
I	1	0.09%	1	0.08%	2	0.17%	0	0%	2	0.02%	0	0%	0	0%
II	42	3.67%	41	3.41%	29	2.44%	24	2.26%	17	1.07%	51	4.75%	49	3.34%
III	681	59.6%	613	51.0%	718	60.4%	654	61.6%	594	59.3%	691	64.3%	923	62.8%
IV	416	36.4%	544	45.2%	435	36.6%	383	36.1%	386	38.6%	328	30.5%	497	33.8%
v	3	0.26%	4	0.33%	4	0.34%	0	0%	2	0.02%	4	0.37%	0	0%
**anemia**	53	4.64%	46	3.82%	44	3.07%	45	4.24%	27	2.69%	32	2.98%	77	5.24%
**Previous acute myocardial infarction**	74	6.47%	89	7.04%	97	8.16%	112	10.6%	105	10.5%	99	9.22%	139	9.46%
**Angina**	97	8.49%	86	7.15%	77	6.48%	72	6.79%	66	6.59%	64	5.96%	116	7.9%
**Chronic obstructive pulmonary disease**	19	1.66%	17	1.41%	15	1.26%	16	1.51%	17	1.07%	17	1.58%	37	2.52%
**Asthma/ bronchitis**	9	0.79%	14	1.16%	6	0.51%	6	0.57%	3	0.3%	1	0.09%	15	1.02%
**Stroke**	18	1.57%	13	1.08%	15	1.26%	9	0.85%	10	1%	13	1.21%	25	1.07%
**Dilated Cardiomyopathy**	15	1.31%	8	0.67%	11	0.93%	11	1.04%	7	0.07%	13	1.21%	19	1.29%
**Cardiogenic Shock**	9	0.79%	8	0.67%	7	0.59%	15	1.41%	18	1.8%	5	0.47%	19	1.29%
**Peripheral arterial disease**	16	1.04%	20	1.66%	14	1.18%	5	0.47%	8	0.08%	7	0.65%	12	0.82%
**Valvar endocarditis**	31	2.71%	23	1.91%	31	2.61%	24	2.26%	20	2%	18	1.68%	35	2.38%
**Cardiac insufficiency^c^**	67	5.86%	69	5.74%	84	7.07%	67	6.31%	48	4.8%	50	4.66%	67	4.56%

a IQR, Interquartile Range 25% and 75%.

b American Society of Anesthesiologists. ^§^Defined as the inability of the heart to provide the necessary blood flow for the vital organs (cardiac failure without ventricular failure, ventricular failure with or without abnormal ventricular systolic function, prolonged tachycardias or tachyarrhythmias in normal hearts).

Details of outcomes and length of hospital stay of the population analyzed between 2013 and 2019 are detailed in Table 2.

**Table 2:** Characteristics of the population analyzed: outcomes and hospital stay.

	2013		2014		2015		2016		2017		2018		2019	
Variables	n	%	n	%	n	%	n	%	n	%	n	%	n	%
**Intra-aortic balloon**	124	10.8%	156	13.0%	113	9.51%	101	9.52%	73	7.29%	55	5.12%	143	9.73%
**Hemotransfusion**	154	13.5%	134	11.1%	161	13.5%	126	11.9%	191	19.1%	307	28.6%	371	25.3%
**Length of stay in the preoperative phase (days), median (IQR)**^a^	5.10	(1.10‒10.5)	3.00	(1.00‒7.40)	3.60	(0.90‒7.40)	2.80	(0.90‒6.90)	2.10	(0.80‒6.50)	1.70	(0.80‒5.80)	1.10	(0.70‒40.7)
**Operating room time (hours), median (IQR)**^a^	6.83	(6.00‒7.75)	6.83	(6.08‒7.67)	6.62	(6.17‒7.83)	7.00	(6.33‒7.95)	7.25	(6.42‒8.25)	7.12	(6.25‒8.17)	6.83	(6.08‒7.67)
**Length of stay in the postoperative phase (days), median (IQR)**^a^	9.25	(7.10‒15.8)	9.10	(7.00‒14.8)	9.00	(7.00‒14.0)	8.10	(6.80‒12.5)	8.80	(6.90‒14.8)	8.00	(6.70‒13.8)	8.90	(6.90‒14.5)
**Length of hospital stay (days) median (IQR)**^a^	17.0	(11.1‒27.6)	15.0	(10.0‒22.9)	14.5	(9.40‒21.9)	13.7	(9.00‒20.4)	13.4	(9.10‒21.7)	12.7	(8.60‒19.9)	12.4	(8.80‒18.9)
**30-day mortality**	94	8.22%	82	6.82%	89	7.49%	69	6.5%	57	5.69%	50	4.66%	46	3.13%

This analysis does not include aortic and congenital surgeries.

a IQR, Interquartile Range 25% and 75%.

### Study design

**Fig. 1:**
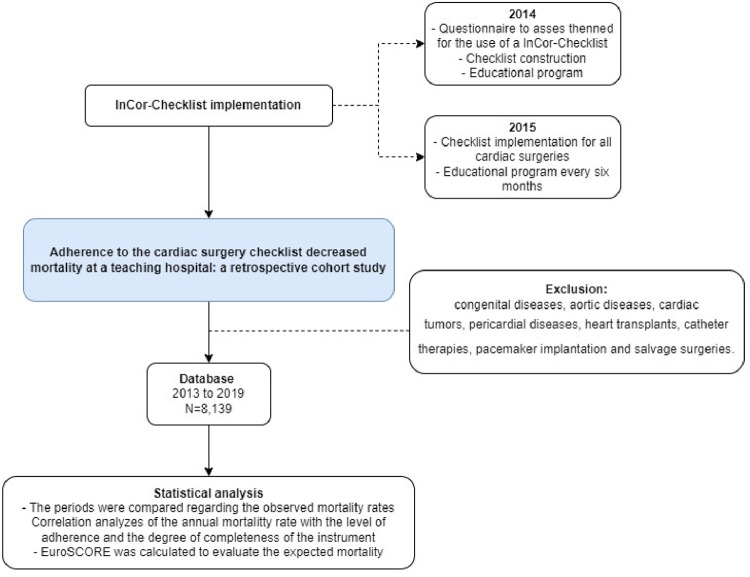
Study design flowchart.

This is a single-center retrospective analysis performed by the Quality and Safety Surgical Unit (UCQSP). The authors complied with the Strengthening the Reporting of Observational Studies in Epidemiology (STROBE) Statement.^10^ In total, 8139 patients were analyzed. It is worth noting that, in 2015, the Continuous Quality Improvement Program in Cardiovascular Surgery (PMCQ) was established. The public and monthly presentation of the results, the monitoring of the mandatory use of the surgical InCor-Checklist, the establishment of the clinical and surgical outpatient clinic, the root-cause analysis of surgical mortality, the development of Quality and Safety Research and the multidisciplinary discussion of the ideal moment to approach emergency patients are among the actions of this program. Therefore, even though the implementation and monitoring of the mandatory use of the InCor-Checklist was the intervention with the greatest impact within the PMCQ, the authors believe that the results may have also been influenced by the other actions oriented during this period. The main outcome was operative mortality defined as when it occurred during the hospitalization in which the operation was performed, even after 30 days; as well as all deaths that occurred after hospital discharge, but before the end of the thirtieth postoperative day. The EuroSCORE II was used as a reference to assess the risk of expected mortality. The flowchart of the study design is shown in Fig. 1. All patients operated on during the study period were included in the analysis and there were no missing regarding the operative mortality.

### Implementation of the InCor-Checklist

In 2014, a questionnaire was applied to assess the need for the use of an InCor-Checklist by the teams (surgeons, anesthetists, perfusionists and nurses) in surgical patients (Table 3), and from this, the InCor-Checklist was built.

**Table 3:** Questionnaire on the need for implementation of the InCor-Checklist (12/08/2014).

Questionnaire	Answer^a^ (mean ± standard deviation)
I believe that the InCor-Checklist will make the surgical team better able to perform safe surgeries	4.86 ± 0.4
I believe that the InCor-Checklist will be easy to use	4.01 ± 0.9
I would use the InCor-Checklist if I was part of the surgical team	4.98 ± 0.1
If I had an operation, I would like the surgical team to use the checklist	4.94 ± 0.2

a Score ranges from 1 (completely disagree) to 5 (completely agree).

**Fig. 2:**
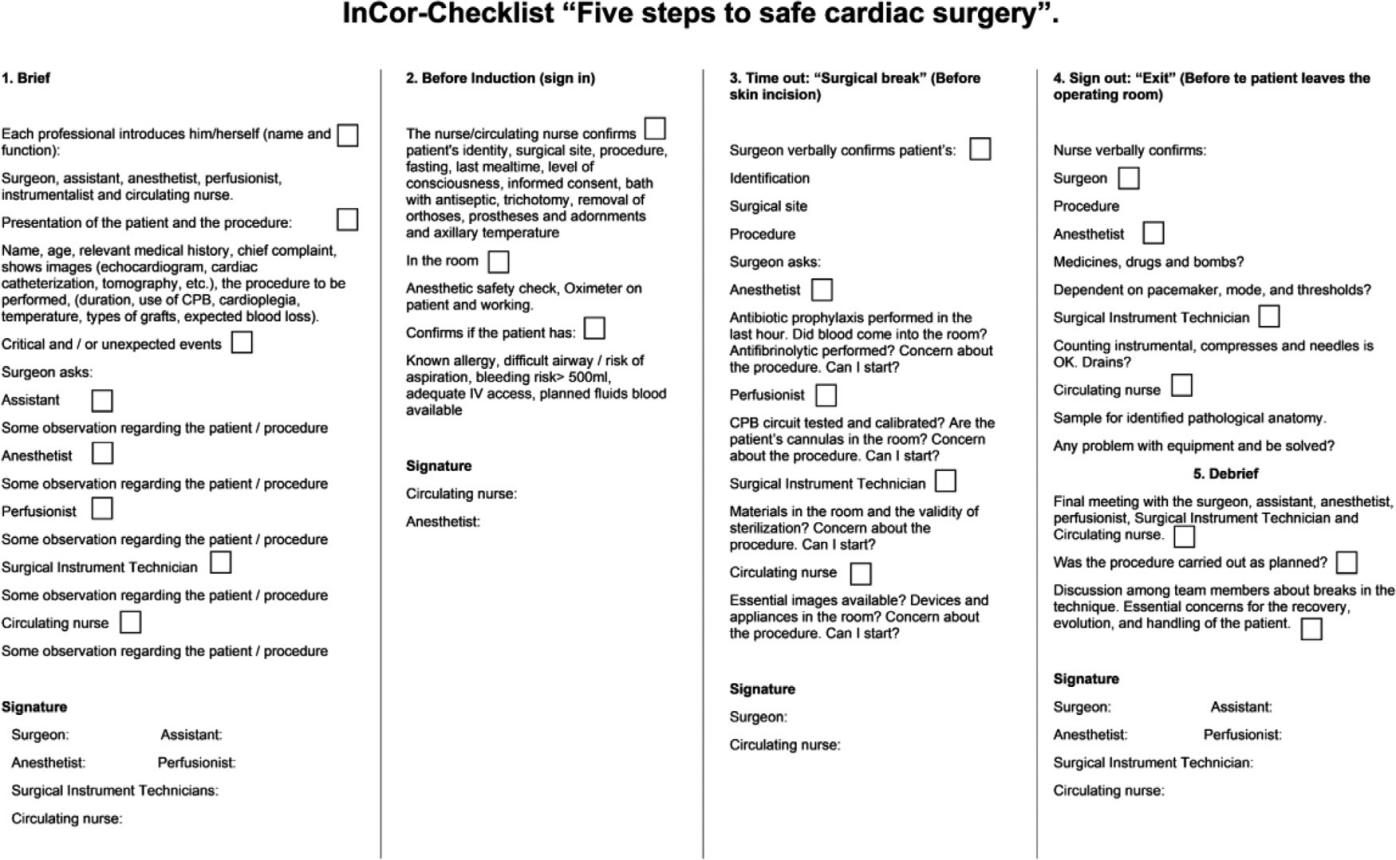
InCor-Checklist “Five Steps to Safe Surgery” form.

This model was established within an educational program composed of standardized classes, teaching materials, videos and simulations in scenarios set up in the operating room. In 2015, the checklist was implemented in the surgical routine to be used in all cardiac surgeries. The idealized model includes 5 sequential phases: Briefing (team planning in relation to the patient and specific surgery), Sign In (before the patient enters the operating room), Time out (before skin incision), Sign out (before the patient leaves the operating room) and Debriefing (report of what happened and how to improve) (Fig. 2). The detailed flow of the InCor Checklist application is shown as supplementary material.

Training with the teams were carried out every six months. Theoretical and realistic simulation classes were included with the purpose of evaluating its applicability, identifying possible adaptations in the process and/or in the instrument, and avoiding the deterioration of the processes.

### Statistical analysis

Regarding the observed mortality, the periods were compared using the two-tailed test to compare proportions. Only the annual average of surgeries performed was analyzed. For the variable number of surgeries, the two-tailed Mann-Whitney test was used. Correlation analyzes of the annual mortality with the level of adherence and the degree of completeness of the instrument were performed. To perform the correlation, Pearson's coefficient was used. The level of significance was set at 0.05. The R software (version 3.5.3) was used for analysis and charts. The Excel program was used to consolidate the original database. As treatment of missing data, the variables “left ventricular ejection fraction”, “CABG time” and “anoxia time” had blank observations filled in with the database mean, similarly, in the analysis of the multiple logistic models as continuous variables were filled in with the observed mean.

### Ethics and consent form

This project was approved by the Ethics Committee for Analysis of Research Projects (CAAE: 4141821.9.0000.0068). The analysis was made from the institution's database and validated by UCQSP without identifying the patients. For this reason, informed consent was waived.

## Results

The mean age of the study population was 60.31±2.09. A total of 5009 (61.54%) patients were male, and 17.16% of the patients underwent urgent surgery. In adults, the most common type of procedure was isolated coronary artery bypass grafting surgery (4146 procedures = 51%).

**Fig. 3:**
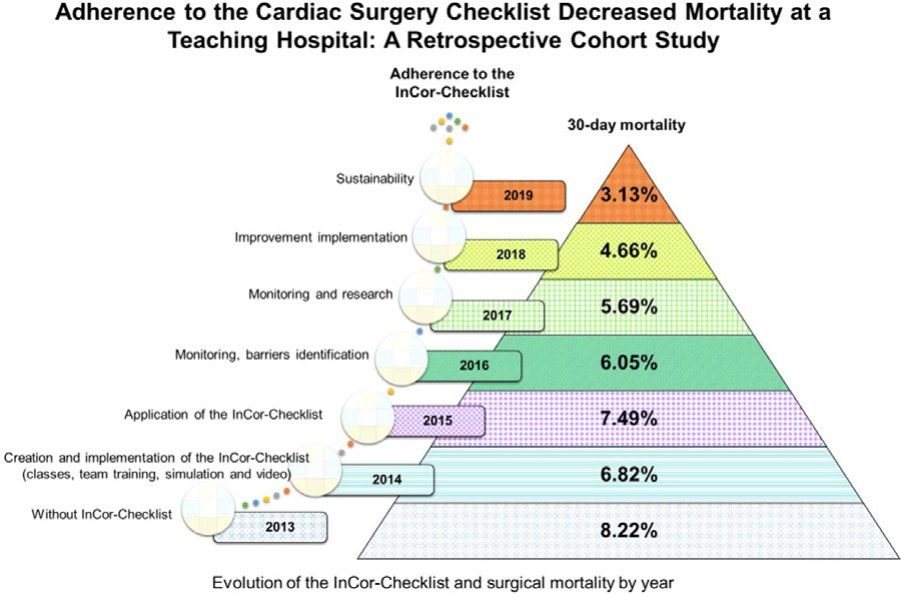
Interventions in the InCor-Checklist use and the association of adherence to the instrument and surgical mortality over time.

The average annual mortality was 5.98% (8.22% in 2013, 6.82% in 2014, 7.49% in 2015, 6.5% in 2016, 5.69% in 2017, 4.66% in 2018, and 3.13% in 2019, as shown in Fig. 3), while the expected mortality calculated by the EuroSCORE was mean 2.54±4.07% (1.98±1.13 in 2013, 1.51±0.53 in 2014, 2.53±2.92 in 2015, 2.72±4.58 in 2016, 2.52±4.22 in 2017, 2.51±4.23 in 2018 and 2.48±4.31 in 2019) When barriers were identified, the processes for the InCor-Checklist use were improved in a cycle of continuous improvement. One of these actions, taken in 2018, was the need to have two surgery representatives in the surgery room to perform the checklist.

Regarding the completeness of the 5 phases of the InCor-Checklist, it evolved from 3.3 in 2015 to 3.9 in 2019 according to sample evaluations year by year (questionnaires). Whenever the InCor-Checklist was used in the operating room, the phases always performed were: Sign-in, Time out, and Sign out, followed by Briefing and finally Debriefing (Table 4). The implementation of the checklist was done gradually among the surgical teams, in a strategic way, aiming to stimulate adherence to the system, avoiding fatigue of those involved in the checking system, until this process became a hospital routine.

**Table 4:** Adherence to the 5 phases of the InCor-Checklist in the analyzed period.

Year	Number of surgeries	Mortality	InCor-Checklist use	Checklist completeness (5 phases)
2013	1143	8.22%	0%	‒
2014	1203	6.82%	0%	‒
2015	1188	7.49%	58%	3.3
2016	1061	6.5%	75%	3.2
2017	1001	5.69%	72%	3.1
2018	1074	4.66%	94%	3.6
2019	1469	3.13%	100%	3.9

∼ Approximately.

Over time, the use of InCor-Checklist for these procedures has been progressive, reaching 75% of all cardiac surgeries in 2016 and 100% in 2019 in an institution that performed an average of more than 100 of these cardiac surgeries per month during this period.

## Discussion

Studies show that adverse events happen more commonly in the operating room (43%) for patients admitted for surgery procedures, and most of them are preventable.^1^^,^^11^ The present study's results show that the use of InCor-Checklist was associated with a 62% reduction in mortality. The InCor-Checklist was adapted from the WHO model^5^ and follows the guidelines of the cardiac surgery societies,^6^ and the American Heart Association,^12^ with the aim of reducing the risk of human errors.

The InCor-Checklist was 37% more effective than the checklist used by the National Patient Safety Program in Scotland,^13^ and less than that achieved by a Boston group in a randomized, multicenter study in which a reduction of 47%,^6^ was seen, both in cardiac and non-cardiac surgeries. On the other hand, another study, also performed in non-cardiac surgeries, in hospitals of Ontario, Canada, did not show significance, with a mortality reduction of only 8.5% before and after implementation.^14^

Perhaps the study most similar to ours in relation to the 5-year period analyzed was conducted in Australia and had a mortality reduction of 23.3% after implementation of the checklist, regardless of the duration of surgery.^15^ An Italian study showed that after implementation there was a 27% decrease in mortality within 90 days after surgery but no difference in relation to the 30-day mortality.^16^

However, most of these studies were done in non-cardiac surgeries. In cardiac surgery, little has been published about the impact of the surgical checklist. Perhaps the most influential study is the one recently published, performed in 7 Dutch hospitals, in which the implementation of a checklist was associated with a 43% reduction in mortality up to 120 days after cardiac surgery.^17^ The decrease in mortality in the present study was associated with the implementation, adherence, and advancement of the 5-year checklist program. The evolution from high mortality values to values similar to those of international reference centers may explain the 58% reduction in mortality.

Regarding the importance of performing a training program, an analysis showed that hospitals in South Carolina (USA) that completed the checklist program (14 hospitals) had a greater reduction in mortality than other hospitals (44 hospitals). There was a 16% mortality reduction before and after implementation of the checklist while there was no difference in the group that did not participate in the program.^18^

As in the present study, adherence to the checklist was also related to leadership involvement, the commitment of the surgical team, and the improvement of communication.^19^ Regarding the impact of completeness of the checklist, a study also showed that there is a lower risk of mortality when the 3 main phases (Sign in, Time out and Sign out) were applied.^20^ Thus, the influence of the use of the checklist was related to risk management before surgery, teamwork, and communication.^6^

Communication mistakes are the most common cause of adverse health events. These mistakes occur because the information does not reach the right person, is inaccurate, or because the problems remain unsolved until they become critical.^21^ Therefore, it would not be simply the implantation of one more questionnaire, but the full conviction that the authors are within a system in which the result will depend on the effective communication of the team and between the teams in the different modalities of assistance to the surgical patient.^22^

The InCor-Checklist includes Briefing and Debriefing within its flow. This was based on a study in which the use of a structured briefing managed to reduce the average number of communication failures by surgery from 3.95 to 1.31 (*p* < 0.001). A structured briefing is associated with an improvement in situational awareness, decision making, teamwork, and the reliability of clinical interventions.^23^^,^^24^

The US Veterans Health Administration training program,^25^ that included briefing and debriefing, in addition to the use of the surgical checklist (Sign in, Time out and Sign out), achieved a 50% greater mortality reduction in the trained group than in the untrained group (*p* = 0.01). Another study also showed that, after adopting briefing and debriefing as a complement, communication improved. This could be the initial step towards a substantial and sustainable organizational transformation.^26^

We believe that the construction of a safety culture for surgical patients at the present institution was positive and progressed in a sustainable manner over 5 years, reaching 100% adherence. The impact of the checklist on the formation of the Safety Culture has been positive in previous studies.^27^, ^28^, ^29^

Considering that approximately 30%‒47% of the complications of the patients admitted to surgery are related to the operating room, a more comprehensive checklist strategy may be ideal. This was assessed by the SURPASS study in the Netherlands and published in NEJM in 2010.^30^ This study not only implemented a perioperative checklist, but also considered all phases of the surgical process and combined it in a uniform way from admission to discharge.

## Limitations

One of the limitations of this study is that it is an observational and retrospective study based on the institutional database. However, all data were validated by the Informatics Service, the Hospital Medical Information Unit, and the Quality and Safety Surgical Unit. The second limitation is that other actions were also included in the same period within the quality improvement program at the institution, the same ones that may have influenced the results of surgical mortality.^31^ For this reason, the authors cannot highlight that there was causality, only association. However, it is undeniable to highlight the correlation between a progressive and sustainable adherence to the checklist and the reduction of mortality. Another limitation of this study is related to the fact that the idealized checklist had no adherence to savage surgery, nor includes crisis situations that may arise during surgeries. A checklist for crisis situations was developed by the Harvard group and resulted in a 6-fold reduction in adherence failure in critical stages in the evaluated scenarios.^32^ The impact of this model on our practice will be assessed in further studies.

It seems that it is not only the technical skill but also the surgeon's behavioral patterns and non-technical skills (leadership, teamwork, problem-solving, decision making, and situational awareness) that affect the surgical results.^33^ A multicenter study on congenital heart surgery in the United Kingdom indicated that the results were not solely related to the technical difficulties of the surgery, as there was a strong relationship between the non-technical skills of the surgeon and adverse events, including death.^34^

Although there are still limitations to the completeness of the 5 phases, the authors can say that the InCor-Checklist achieved your goal: the formation of the safety culture of the surgical patient. Thus, the authors can see that the implantation and sustainability of a surgical checklist are not as simple as it seems; it requires a lot of leadership, humility, and teamwork.^35^

## Conclusion

In the formation of the surgical patient safety culture, the implementation and adherence to the InCor-Checklist “Five steps to safe cardiac surgery” was associated with decreased mortality after cardiac surgery. The authors recommend that hospitals, in addition to implementing a surgical checklist, should develop strategies to encourage adherence and completeness, as well as sustainability within public policies.

## Author's contribution

OAVM and FBJ conceived, designed, and directed the project. OAVM, FCCM, and MP were involved in planning and supervising the work. FCCM, LABNS, FRBGG, LFC, and JFMF Collected the data. LRPD and LAFL performed the analysis, drafted the manuscript, and designed the figures. OAVM aided in interpreting the results and wrote the manuscript. All authors discussed the results and revised the final content of the manuscript.

## Funding

This research did not receive any specific grant from funding agencies in the public, commercial, or not-for-profit sectors.

## Conflicts of interest

The authors declare no conflicts of interest.
